# Experience and generalization in a connectionist model of Mandarin Chinese relative clause processing

**DOI:** 10.3389/fpsyg.2013.00767

**Published:** 2013-10-22

**Authors:** Yaling Hsiao, Maryellen C. MacDonald

**Affiliations:** Department of Psychology, University of Wisconsin-MadisonWI, USA

**Keywords:** Simple Recurrent Networks, relative clauses, sentence processing, Mandarin Chinese, working memory, connectionism

## Abstract

Sentences containing relative clauses are well known to be difficult to comprehend, and they have long been an arena in which to investigate the role of working memory in language comprehension. However, recent work has suggested that relative clause processing is better described by ambiguity resolution processes than by limits on extrinsic working memory. We investigated these alternative views with a Simple Recurrent Network (SRN) model of relative clause processing in Mandarin Chinese, which has a unique pattern of word order across main and relative clauses and which has yielded mixed results in human comprehension studies. To assess the model's ability to generalize from similar sentence structures, and to observe effects of ambiguity through the sentence, we trained the model on several different sentence types, based on a detailed corpus analysis of Mandarin relative clauses and simple sentences, coded to include patterns of noun animacy in the various structures. The model was evaluated on 16 different relative clause subtypes. Its performance corresponded well to human reading times, including effects previously attributed to working memory overflow. The model's performance across a wide variety of sentence types suggested that the seemingly inconsistent results in some prior empirical studies stemmed from failures to consider the full range of sentence types in empirical studies. Crucially, sentence difficulty for the model was not simply a reflection of sentence frequency in the training set; the model generalized from similar sentences and showed high error rates at points of ambiguity. The results suggest that SRNs are a powerful tool to examine the complicated constraint-satisfaction process of sentence comprehension, and that understanding comprehension of specific structures must include consideration of experiences with other similar structures in the language.

## Introduction

Sentence comprehension is generally considered to be a complex constraint satisfaction process integrating probabilistic information from syntactic, semantic, prosodic, and discourse sources (e.g., MacDonald et al., 1994; Tanenhaus and Trueswell, 1995). This emphasis on multiple probabilistic constraints in sentence comprehension demands precise accounts of how constraints of different types and different strengths are weighed, so as to yield clearly testable models of comprehension. Unfortunately, compared to a large number of empirical studies in sentence comprehension, there are relatively few implemented computational models of sentence processing phenomena, which could illuminate the interaction of complex probabilistic constraints in sentence comprehension.

One issue that has been addressed in computational models of sentence comprehension is the role of computational capacity in accounts of human comprehension behavior. In particular, a key question in comprehension research is the separability between linguistic knowledge and the capacity to use that knowledge in comprehension and other language behavior. These issues are familiar in the competence-performance distinction that has traditionally distinguished much of linguistic and psycholinguistic research (e.g., Miller and Chomsky, 1963), but it also arises within psycholinguistic accounts of complex sentence comprehension—how much is human comprehension difficulty attributable to limitations on human working memory capacity, independent of people's experience with language? For example, several different accounts have been offered for the difference in comprehension difficulty for subject relative clauses and object relative clauses in English and many other languages. An example can be seen in sentences (1a–b), where the object relatives (1b) are generally found to be more difficult than subject relatives (1a).

1. Subject Relative Clause: The candidate **[who_1_ attacked_1_ the opponent]** won this election.
2. Object Relative Clause: The candidate **[who_1_ the opponent attacked_1_]** won this election.

A common argument for the difference in comprehension difficulty between these two sentence types points to the role of working memory, that the object relatives (1b) place higher working memory demands on the comprehender than do the subject relatives (e.g., King and Just, 1991). In one variant of this view, the Dependency Locality Theory (Gibson, 1998), the working memory demands are tied to greater distance between related elements (shown with subscripts in 1a–b) in object relatives (1b) compared to subject relatives (1a). This additional distance in (1b) requires longer memory maintenance of the partially processed information (“the candidate”) until it can be integrated with the action (“attacked”). Failure to maintain or retrieve the information disrupts comprehension. Thus, on this view, comprehension difficulty is directly tied to the capacity to maintain discontinuous elements during sentence comprehension.

**Figure 1:**
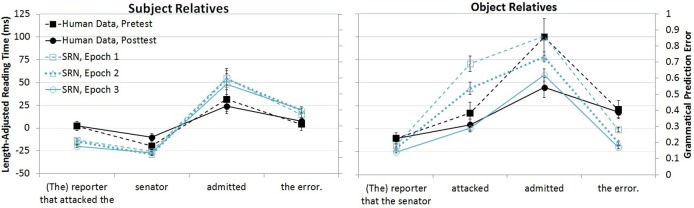
**Comparison of SRN performance in MacDonald and Christiansen (2002) and human reading times in Wells et al. (2009)**.

These questions have also been addressed in computational models of sentence processing. Simple Recurrent Network (SRN) models of sentence comprehension have a computational capacity that is inherently tied to the model's experience with linguistic input (MacDonald and Christiansen, 2002; this is true of connectionist models more generally, e.g., McClelland and Elman, 1986). As originally implemented by Elman (1990), an SRN is a partially recurrent network equipped with an additional context layer that stores the output of the hidden layer, which is paired with the next input to the network. The model's task is to predict the next input word, and the presence of the context layer permits the prior linguistic context to influence the prediction of later words. SRNs have been applied to several different types of complex sentences (e.g., Elman, 1991, 1993; Christiansen, 1994; Christiansen and Chater, 1999), including the contrast in difficulty between subject and object relatives, as in (1a–b). Direct comparisons of human and model performance reveal important similarities. Figure 1 shows MacDonald and Christiansen's (2002) SRN and Wells et al's. (2009) human subjects' reading times for subject and object relatives in studies in which experience with the two structures was explicitly manipulated in the model and in the human readers. MacDonald ad Christiansen's model had extensive experience with several kinds of simple sentences, while only 5% of the sentences in the training set contained relative clauses. Their model was tested at three different points in training to investigate a claim about how these models generalize from the common simple sentences, such as *The candidate attacked the opponent*, to relative clauses such as (1a–b). They hypothesized that interpretation of subject relatives would be aided by these sentences' similarities to simple sentences, and as a result, the model would rapidly learn to make accurate predictions for subject relatives, and it would show little effect of additional training, as it had already benefited from the overlap with the common simple sentence “neighbors.” By contrast, object relatives have an idiosyncratic word order that is not aided by extensive experience with simple sentences, and MacDonald and Christiansen predicted that as a result, the model would be extremely sensitive to the degree of direct experience with object relatives. Figure 1 shows that these predictions were supported, and it also shows that human reading times in the Wells et al. study were similarly influenced by a training manipulation, in which additional exposure to subject and object relatives over the course of a month affected participants reading patterns for object relatives (right panel) but not subject relatives (left panel).

An important feature of SRNs is that they generalize over their training experiences with individual sentences to find regularities across the training items (Elman, 1990; St John and McClelland, 1990). The results in Figure 1 reflect generalization in MacDonald and Christiansen's SRN—in that object and subject relatives were presented equally often but were not equally difficult, because the model generalized from simple transitive sentences to the similar subject relatives. Several other researchers have pursued this point, including Fitz et al. (2011), who used a dual-path SRN that models both sentence production and sentence semantics. Although these models are not meant to be accounts of the acquisition process, similar generalization effects have been found in child language acquisition. For example, Yip and Matthews (2007) found that the relative clauses that emerged earlier in Cantonese (object relatives) do so because of their word order resemblance to the dominant word order in simple sentences (for similar effects in other languages, see Diessel, 2004, 2007; Diessel and Tomasello, 2005; Ozeki and Shirai, 2007).

These results hold promise for SRN accounts of sentence comprehension, but to date, their application has been quite limited, and in particular the models have not typically incorporated realistic frequencies of various sentence types, which would better allow researchers to understand how complex probabilistic constraints are weighed in comprehension. In this paper, we take steps toward meeting this challenge with an SRN model of relative clause comprehension that accurately represents critical elements of the distributional knowledge that humans bring to bear in interpreting these structures. Rather than elaborating accounts of English relative clause comprehension, about which an enormous body of research exists, we address relative clause comprehension in Mandarin Chinese. As we detail below, Mandarin relative clause comprehension is particularly interesting because (a) the relative clause structure is quite different from English, (b) some key findings about comprehension difficulty show potentially opposite patterns than in English and in many other languages, (c) there is a fair amount of controversy concerning comprehenders' performance in various structures and discourse contexts, and (d) the number of important factors affecting comprehension appears to be too large to be manipulated together in empirical studies. Thus, a computational account of Mandarin relative clause processing has the opportunity to have a substantial impact informing the nature of constraint satisfaction processes in Mandarin and more generally.

### Mandarin relative clauses

Relative clauses in English and many other languages are “head-first,” meaning that the relative clause appears after the head noun, as in *the candidate [that attacked the opponent]*. In many of such languages, comprehenders show a clear pattern of finding subject relatives easier than object relatives (English: e.g., King and Just, 1991; Gibson et al., 2005; Dutch: e.g., Frazier, 1987; German: e.g., Schriefers et al., 1995; French: e.g., Frauenfelder et al., 1980). Other languages, including Korean, Japanese, and Mandarin, have a “head-final” relative clause structure, such that the relative clause precedes the head noun, as in the Mandarin examples in (2). In these examples, in which the relative clause modifies the subject of the main clause, the relative clause begins the sentence, ending with the relativizer DE (equivalent to the English *that* in this context), followed by the noun phrase (head) being modified, in this case *candidate*. This head-final relative clause word order is a critical factor in accounts of cross-linguistic differences in relative clause processing, because the different relative clause structures create different degrees of distance between dependent elements, as shown by subscripts in (2).

Subject-modifying subject relative: 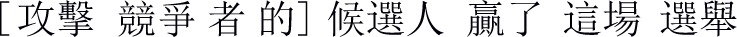  [*e*_1_ attack opponent DE] candidate_1_ won this election The candidate who attacked the opponent won this election.
Subject-modifying object relative: 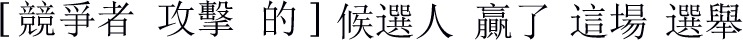  [opponent attack *e*_1_ DE] candidate_1_ won this election The candidate who the opponent attacked won this election.

Most studies of relative clause processing in Korean and Japanese have suggested that subject relatives are easier than object relatives (Japanese: e.g., Miyamoto and Nakamura, 2003; Korean: Kwon et al., 2010), similar to English and other head-first languages. Some researchers suggest that this pattern points to a universal subject preference for relative clause processing, as first proposed by Keenan and Comrie under the term “Accessibility Hierarchy” (1977), which argues that noun phrases at the subject position are the easiest to be relativized due to their higher syntactic position, compared to noun phrases lower in the hierarchy, such as genitive (though cf. Ozeki and Shirai, 2007; Yip and Matthews, 2007). However, the pattern of subject preference does not appear to apply to Mandarin, which differs in several ways from the other languages considered here. Mandarin has the head-final relative clause structure, like Japanese and Korean, but whereas Japanese and Korean have a rich system of case marking on nouns that presumably aids relative clause processing, case-marking is non-existent in Mandarin. Mandarin is also different in that it has the dominant word order of subject-verb-object (SVO) in main clauses, like English and many European languages, but with the head-final relative clause structure that is absent in these languages. This combination of SVO basic word order, head final relative clause structure and absence of case marking is attested in the world's languages only in four Sino-Tibetan languages such as Bai, and other Chinese languages like Mandarin, Cantonese, and Hakka (Keenan, 1985; Haspelmath et al., 2005).

These features make Mandarin an interesting test case to disentangle the influences of various potential factors in relative clause processing. To date, empirical studies have yielded conflicting results, with some studies finding the typical cross-linguistic pattern of subject relatives being easier than object relatives (e.g., Lin and Bever, 2006), and others finding the opposite result (e.g., Hsiao and Gibson, 2003). This reversal of the dominant cross-linguistic pattern finds a clear interpretation in the Dependency Locality account. Hsiao and Gibson argued that subject relatives (e.g., 2a) were more difficult than object relatives (e.g., 2b) because they have higher storage and integration costs, owing to the longer distance between dependencies in subject relatives: there are more intervening words between the filler and the gap and thus more new discourse referents and incomplete dependencies in a subject relative than in an object relative. Support for this memory-based view comes from a study in which the added difficulty of subject relatives was higher in participants with lower working memory span (Chen et al., 2008) and patients with aphasia (Su et al., 2007). Several studies manipulating other factors, such as relative clause topicalization (Lin and Garnsey, 2011) and context (Gibson and Wu, 2013), also found a similar object relative advantage.

However, a reading time advantage for subject relatives has also been reported in several studies. Vasishth et al. (2013) modified Hsiao and Gibson (2003)'s materials and failed to replicate their effect, instead finding that object relatives were harder than subject relatives. Lin and Bever (2011) found no difference between the two types of relative clauses modifying the main clause subject (as in 2a–b) but found shorter reading times for subject relatives than object relatives when the relative clause was modifying the main clause object, such that subject relatives like (3a) were easier than object relatives like (3b). They also manipulated whether the participants were informed that they were reading relative clauses and about which noun positions they were modifying. They found that participants who were not informed had the most difficulty reading object-modifying ORCs, whose word order in combination with the matrix clause (i.e., the first three words in 3b) could lead the readers into a garden path of a simple sentence.

Object-modifying subject relative: 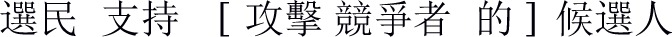  voter support [*e*_1_ attack opponent DE] candidate_1_ Voters support the candidate who attacked the opponent.
Object-modifying object relative: 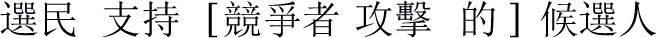  voter support [opponent attack *e*_1_ DE] candidate_1_ Voters support the candidate who the opponent attacked.

Still other studies investigated the effect of lexical semantics and found it to have a modulating influence on the difficulty of the two relative clause types. Wu et al. (2012) manipulated the animacy of both the head noun and the relative clause noun and found that comprehension difficulty in both sentence types was strongly dependent on whether the sentence contained a canonical animacy configuration, in which animate entities acted on inanimate ones. These results resonate well with the Mak et al. (2002, 2006) and Traxler et al. (2002, 2005) studies in other languages, in that animacy serves an important cue for thematic role assignment, which in turn affects ambiguity resolution (Gennari and MacDonald, 2008).

More generally, these results, together with absence of case marking and head-final relative clause structure in Mandarin, suggest that difficulty in relative clause comprehension may be strongly modulated by temporary ambiguities in sentences, where comprehenders initially interpret nouns and verbs in the input as being part of a main clause only to realize later that they had encountered a relative clause.

Summarizing over these various studies of Mandarin relative clause processing, it is impossible to draw conclusions about overall comprehension difficulty of the two sentence types. The inconsistency across studies and the sensitivity of the results to multiple factors such as animacy and modifying position may suggest that comprehenders are able to use very detailed information about patterns of relative clause use in comprehending these structures. Thus, while many prior studies argued for one structure being absolutely easier than the other, a closer look at the materials in these studies suggests researchers' conclusions are limited by their methodological choices and stimulus items. For example, while claims about relative clause processing difficulty are typically phrased to cover all relative clauses of a certain type (such as subject relatives), in fact, most of the previous studies examined only a narrow subset of relative clauses within a given type. For example, most studies have examined only relative clauses that modified main clause subjects, and containing only animate head nouns and relative clause nouns. Such relative clauses are in reality rare in the linguistic environment (Pu, 2007; Wu et al., 2012; Vasishth et al., 2013). As a result, the Mandarin relative clause research, which could in principle be extremely informative about cross-linguistic regularities and differences in complex sentence interpretation, is instead marginalized by a lack of consensus and by overly-broad claims based on a narrow range of stimulus materials.

While is effectively impossible to combine all the potentially important factors in a single empirical study that would allow us to observe complex interactions among them, it is possible to examine many of these effects in an SRN. We conducted our study in two phases, reflecting the fact that an attractive property of SRNs is their strong sensitivity to the distributional patterns in the language, a property that also appears in human comprehension. As described in Study 1, we examined the distributional statistics of relative clauses in a large corpus, extensively hand-coding the corpus data for features we believe to be critical in relative clause comprehension. In Study 2 we used this distributional information to develop a finite state grammar from which we developed training materials that faithfully represented key properties of relative clauses identified in the corpus. We used these training materials to train an SRN that was exposed to several different types of simple sentences and relative clauses. Model performance was then compared to human reading time data from previous studies. Because the corpus analysis and, therefore, the training set, were so detailed, we can compare experiment findings with model performance specifically for the types of sentences used in several empirical studies. In this way, we aim to use the model to help resolve the conflicting findings in the literature and be able to identify broader themes in Mandarin relative clause processing and cross-linguistic differences and commonalities in sentence comprehension more generally.

## Study 1—corpus analysis

Humans' comprehension of relative clauses is influenced not only by their prior experience with relative clauses but also by their experience with other sentences in the language. For example, MacDonald and Christiansen (2002) suggested that English speakers' experiences with simple transitive sentences aided comprehenders' interpretation of subject relative clauses, which are similar in that nouns in analogous sentence positions receive the same thematic role assignments. In this sense simple transitive sentences are helpful “neighbors” of subject relatives in English, as experience with these highly frequent simple sentences allows generalization to the rare subject relative structure (see also Fitz et al., 2011). MacDonald and Christiansen found a similar neighborhood effect in their SRN, attributable to the overlap in word order between the two sentence types, as the SRN does not assign thematic roles or interpret sentences. Conversely, humans' prior experiences with other kinds of sentences can increase the difficulty of relative clause comprehension. Gennari and MacDonald (2008) showed that object relative clauses in English contain temporary ambiguities for which the object relative clause is often an infrequent and disfavored interpretation. In this case, experience with other, more frequent sentence types affects the process of ambiguity resolution, leading to difficulty in interpreting object relatives. We will call these alternative interpretations “competitors,” recognizing that both neighbor and competitor effects reflect comprehenders' generalizations over their prior linguistic experiences.

Mandarin relative clauses may similarly be affected by competitors and neighbors. First, Mandarin head-final relative clauses exhibit temporary ambiguities such that the unfolding linguistic input has several alternative interpretations. Second, certain Mandarin relative clauses have highly similar word orders to some more frequent simple sentences; generalization over these common neighbor sentences should help relative clause processing. Note that the same sentence type may serve both competitor and neighbor functions at different points, in that it might provide an alternative interpretation that affects ambiguity resolution early in processing but that following a point of disambiguation, certain overlap with a relative clause may help in eventual relative clause interpretation (see Fitz et al., 2011, for further discussion). Appendix A describes some of the main neighbors and competitors for relative clauses in Mandarin.

To investigate the range of competitors and neighbors of Mandarin relative clauses, which should be informative for both human and computational work, and to develop a realistic training corpus for our model, we conducted a corpus analysis that enabled us to calculate the statistics of various types of relative clauses, crucial competitor structures that could increase comprehension difficulty, and neighboring structures that could reduce comprehension difficulty.

Here it is necessary to introduce some clarifying terminology, because it can be confusing to discuss both subject and object relative clauses (in which the modified head noun is the subject or object of the relative clause verb, respectively) crossed with the main clause subject vs. object modifying positions (in which the relative clause-modified noun is either the subject or object of the main clause; see examples 2a–b and 3a–b above). For the remainder of this paper, we will refer to relative clauses as RCs, and the subject and object relatives as SRCs and ORCs, respectively. We will continue to spell out subject- vs. object-modifying positions in the main clause, so that full word descriptions are associated with main clause factors and acronyms are used for the embedded clauses.

Table 1 shows the competing and neighbor structures that we investigate in the present study and the ambiguities or facilitation they create at different points of an SRC and an ORC. The cells with gray shading indicate sentence types in the training set for which we do not expect large effects on RC processing in the model. As the table shows, some sentences may serve both competitor and neighbor functions at different points in the sentence.

**Table 1:** **The competing and neighbor structures (listed on the top) we examined in the current study and the ambiguities and facilitation they created at different points for SRCs and ORCs at the two matrix positions (listed on the left)**.

		**Overt subject simple sentences: N V {N}**	**Pro-drop simple sentences: V {N}**	**Subject-modifying intransitive SRCs: [V DE] N V …**	**Object-modifying intransitive SRCs: N V [V DE] N.**
Subject-modifying	SRCs: [V N DE] N V …		Competitor: before DE neighbor: after DE (for V N word order)	Neighbor: early (promotes RC interpretation of first V)	
	ORCs: [N V DE] N V …	Competitor: before DE (interpret the initial N V order as start of simple sentence) neighbor: after DE (similar N V N order)			
Object-modifying	SRCs: N V [V N DE] N.				Neighbor: early (promotes RC interpretation of first V)
	ORCs: N V [N V DE] N.	Competitor: from beginning (interpret RC N as object N of simple sentence)			

N, noun, V, verb, DE, relativizer, SRC, subject relative clause, ORC, object relative clause, [Square brackets] indicate the relative clause, {Curly brackets} indicate optional direct object N, which is present following transitive verbs and absent following intransitive verbs.

Note: Because the model did not include those verbs that can be both transitive and intransitive (e.g., “I ate” vs. “I ate an apple”), intransitive SRCs were not listed as a competitor to transitive SRCs.

### Methods

We used Tgrep2 1.15 (Rohde, 2005) to extract sentences from a parsed corpus of spoken and written language, the Penn Chinese Treebank 7.0 (Xue et al., 2010). The corpus consists of more than one million words in more than 50,000 sentences, with sources from Chinese newswire, broadcast news, magazine news, broadcast conversation programs, web newsgroups, and others. The Tgrep2 search patterns used to extract the sentences are contained in Appendix B. Our aim was to retrieve every subject- and object-modifying ORC, every subject- and object-modifying SRC (with both transitive and intransitive verbs), every single-clause pro-drop construction, and every overt subject single-clause simple sentence in the corpus. A total of 6255 sentences were extracted.

We limited extraction of simple sentences to those with only a single clause, because our model was not designed to process other types of multi-clause sentences. However, we extracted all RCs that modified a sentential subject or object regardless of whether the rest of the sentence was a simple main clause or a more complex sentence. We cast this wider net for RCs because they are fairly rare in Mandarin, and extracting literally every RC in the corpus would give us the best estimate of the relative frequency of different RC types and animacy configurations. These methodological choices yielded a higher ratio of RC sentences to non-RC sentences than in the whole corpus (because many other non-RC multi-clause sentences were not extracted). Nonetheless, simple sentences outnumbered RCs at about 5:1 in the extracted set of 6255 sentences.

For each sentence, noun animacy was coded by hand, as NP animacy is known to affect Mandarin RC processing (Wu et al., 2012), and we expected that the animacy configuration of simple sentences could also serve as important experience for RC processing. Coding followed the criteria that animate nouns refer to living entities that possess agency and volition to perform an action, while inanimate nouns refer entities without these properties (Hundt, 2004). For example, plants are living things but do not have volition, and thus they were coded as inanimate. Nouns that represented a group of people, such as organizations, countries, etc. (e.g., *the school that taught me math*), were considered animate. However, when these nouns were used purely as locations (e.g., *the school that I went to*), they were coded as inanimate. Coding was performed by two native speakers of Mandarin who were instructed in these coding criteria.

### Results

Table 2 reports the frequencies of all simple sentences that were coded, and Table 3 reports all relative clauses. Inspection of these tables reveals the following patterns:

1. Simple sentences with an overt subject (a potentially helpful neighbor of ORCs) (*N* = 3309) were more frequent than pro-drop simple sentences (a competitor for some RC interpretations, *N* = 1885), and sentences with RCs (*N* = 1061) were less frequent than either of these simple sentence types. Thus, both some competitor interpretations and helpful neighbor interpretations are more frequent than RCs themselves.
2. The majority of (overt) main clause subjects were animate and the majority of main clause objects were inanimate. As main clauses are more common than RCs, these patterns of main clause animacy may influence expectations for RC animacy configurations.
3. There were relatively more subject-modifying RCs (*N* = 636, 60% of all RCs) than object-modifying RCs (*N* = 415). SRCs (transitive and intransitive combined) occurred more often than ORCs in both modifying positions. Transitive SRCs were the most frequent among the three types of RCs.
4. ORCs, regardless of modifying position, mostly had inanimate head nouns and animate RC embedded nouns, consistent with patterns observed in English (Roland et al., 2007). Transitive SRCs had a higher proportion of animate heads and inanimate RC embedded nouns in the subject-modifying position, whereas there was not a big difference in head animacy but a preference of inanimate RC embedded nouns in the object-modifying position. Intransitive SRCs at both modifying positions had more inanimate heads than animate heads. These relatively high rates of inanimate head nouns, which generally followed the patterns of animacy usage in main clauses, could be attributed to the high percentage of sentences like “The growth rate rose,” in the newswire genre, which comprises a large subset of the Chinese Treebank 7.0 corpus.

**Table 2:**
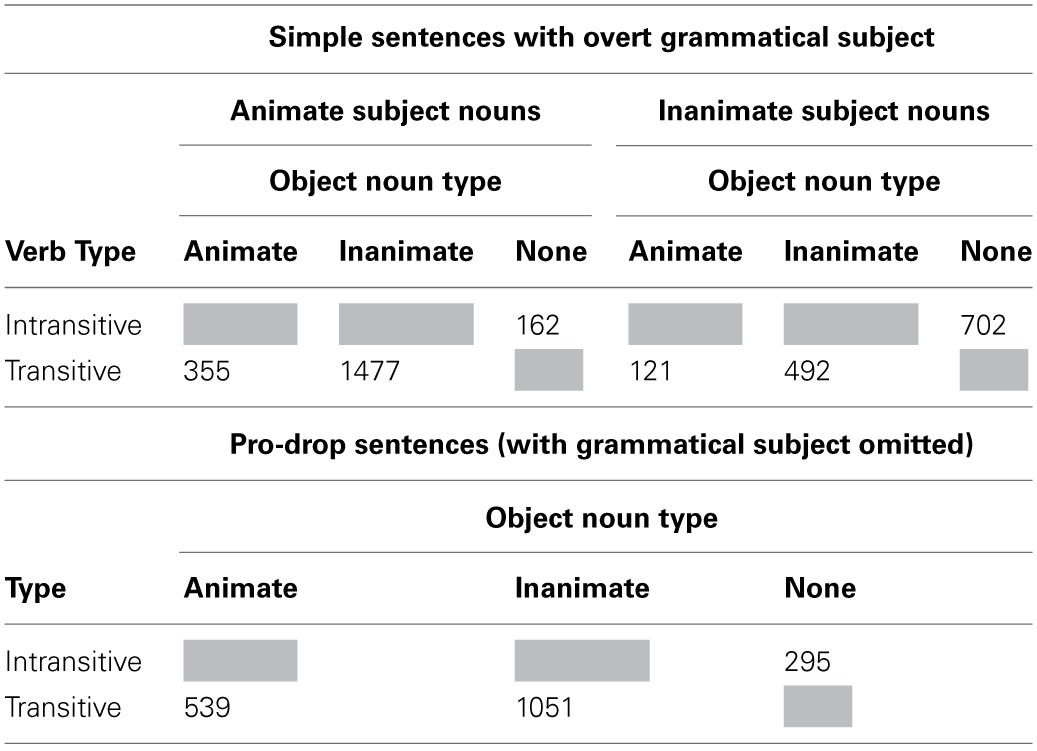
**Token frequencies of overt subject simple sentences and pro-drop sentences found in Chinese Treebank 7.0**.

Gray cells mark non-existent combinations of verb transitivity and object type; for example, intransitive verbs by definition have no direct objects, and so cells representing the animacy coding of objects are not relevant for intransitive verbs.

**Table 3:**
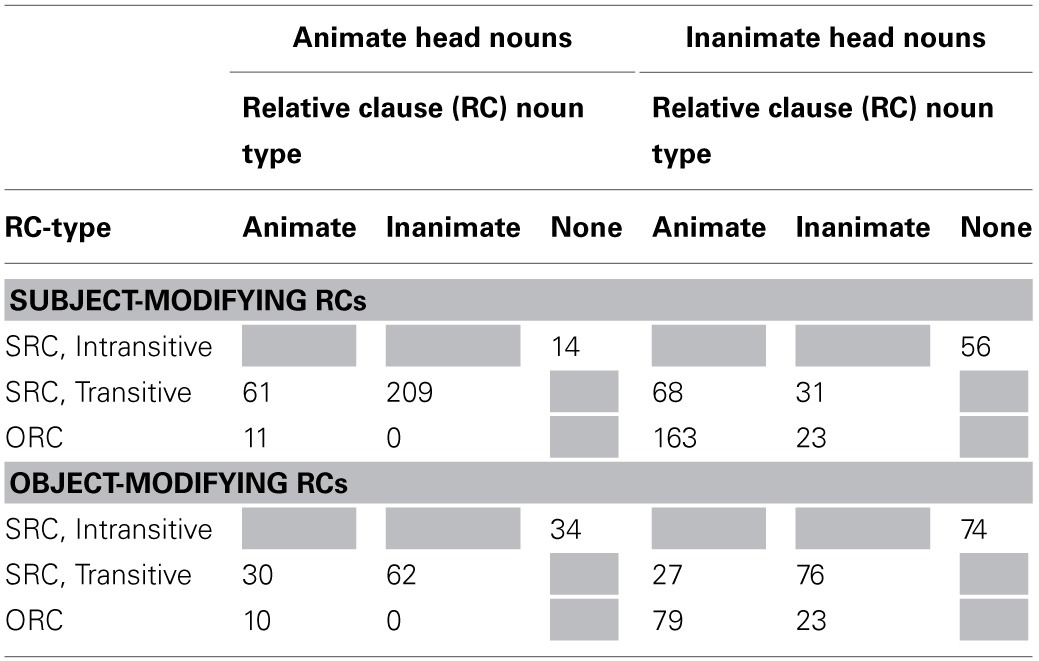
**Token frequencies of subject- and object-modifying SRCs (transitive and intransitive) and ORCs at found in Chinese Treebank 7.0**.

Relative Clause Nouns are the Relative clause object in SRC Transitive sentences and the Relative clause subject in ORC sentences. Gray cells reflect non-existent sentence types (because transitive verbs must have a relative clause noun and intransitive verbs do not have a direct object noun).

### Discussion

This corpus analysis yielded several important patterns. First, the configuration of animacy in the corpus, in both main clauses and relative clauses, is strikingly similar to findings in other languages and also replicates and extends previous Mandarin corpus studies (Pu, 2007; Wu et al., 2012). Key results here include the tendency for main clause subjects to be animate and objects to be inanimate, the tendency of SRC heads to be animate and ORC heads to be inanimate, and the rarity of RCs with two nouns of the same animacy (both animate or both inanimate, Wu et al., 2012). The general similarity of these patterns to main and relative clause usage in other languages (e.g., Bock and Warren, 1985; Roland et al., 2007; Gennari and MacDonald, 2009) suggests that the difference in patterns of relative clause interpretation in Mandarin vs. other languages does not lie in different animacy configurations and must instead reflect other critical cross-linguistic differences. Candidates for other important cross-linguistic differences include the very high degree of temporary ambiguity encountered during Mandarin RC processing, owing to the combination of head-final RCs and the absence of case marking, and also particular patterns of potentially helpful neighboring structures. The complexity of the potential interactions here is quite large, and in the next study, we use a SRN to explore the effect of these linguistic patterns on relative clause processing.

## Study 2—simple recurrent network

We chose an SRN to model Mandarin RC processing because of its potential to simulate word-by-word human reading times, including in prior studies of relative clause processing (MacDonald and Christiansen, 2002; Wells et al., 2009; Fitz et al., 2011). We trained the model on a mix of relative clauses, helpful neighbor sentences, and competitor sentences (contributing to temporary ambiguities) in proportions based on the Study 1 corpus analysis to investigate how these varied experiences could jointly contribute to relative clause processing. It is important to note that SRNs are not simulating human language comprehension *per se* but are instead a simulation of a component thought to be a part of comprehension and a factor related to reading times, namely prediction of upcoming input. Thus, while terms such as “ambiguity resolution,” “alternative interpretations,” and “garden-path effect” are common in descriptions of human reading times, the model is not adopting any interpretation or calculating meaning. Ambiguity created by the existence of multiple interpretations in human sentence processing is more adequately termed as “conditional indeterminacy” in the case of an SRN. As the probabilities of grammatical continuations are more varied, the higher the prediction error is for the model. Thus, for both model and humans, indeterminacy is costly, and the model is taken to represent one important aspect of the ways in which input can be ambiguous for humans.

### Methods

The SRN used the backpropagation learning algorithm. It contained a context layer in addition to input, output and hidden layers. These layers were connected by trainable weights, except that the context layer directly copied the activations of the hidden layer from the previous time tick. The input pattern (the words of a sentence) is activated in the input layer one word at a time and then propagated onto the hidden layer and the output layer at time step *t*. The weights are adjusted by comparing the output activations to the desired output. At the next time step *t* + 1, the output activation on the hidden layer at time step *t* is copied to the context layer and then projected backed to the hidden layer to pair with the current input.

#### Model specification

**Figure 2:**
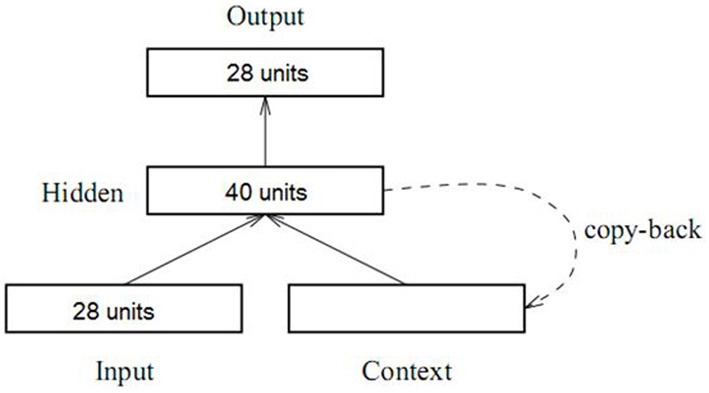
**Architecture of the Simple Recurrent Network used in Study 2**.

As shown in Figure 2, the model contained 28 localist units in the input and output layer, representing 27 words, plus one “end-of-sentence” marker. Although the network did not model semantics, animacy was captured distributionally, meaning that units designating “animate” nouns appeared frequently as sentence subjects and rarely as objects, while units designating inanimate nouns had the opposite pattern. The grammatical categories included in this model were the following: Animate Nouns (7 units), Inanimate Nouns (7 units), Transitive Verbs (6 units), Intransitive Verbs (6 units), RC marker DE (1 unit), and End-of-Sentence marker (1 unit). There were 40 units each in the hidden layer and the context layer. The learning rate was set to 0.05, with momentum of 0.9 and the batch size of 1. The simulation was conducted with the software Lens (Version 2.63) (Rohde, 1999).

#### Training

Based on the frequency information obtained from Study 1, we calculated the bigram transitional probabilities from one grammatical category to another, as shown in Table 3. Transitional probabilities were calculated from the probability of occurrence of Y given the previous input X. For example, given the occurrence of a transitive verb (VT), the next word was an animate noun (aniN) 18% of the time, an inanimate noun (inaN) 73% of the time and the start of an object modifying relative clause (objRC) 9% of the time. Similarly, the probability of the occurrence of a subject-modifying SRC with an animate head and intransitive verb is 0.7 × 0.16 × 0.11 × 0.2 = 0.0025, meaning that there are ~25 sentences that contain this type of RC in the 10,000-sentence training corpus.

The model's training faithfully reflected the relative frequencies of RCs found in the corpus, but as with all computational models, it is a simplification of the knowledge that humans bring to the task. These simplifications include the absence of semantics, the limited vocabulary, the omission of other uses of DE (see Appendix A), and having a higher proportion of RCs in the training set than in the corpus as a whole. Some of these features (RC frequency, definitive disambiguation at DE) may make RCs proportionally less difficult for the model than for humans, and other features (absence of fine grained semantics or real world or discourse context, smaller neighborhood effects) could make RCs yield proportionally more error for the model than would be expected based on human reading times. Given that all models necessarily include simplifications, the choices here represent a good starting point with which to examine Mandarin RC processing.

A python script was written based on the finite state grammar with the transitional probabilities in Table 4 to produce the 10,000 training sentences. The grammar did not permit sentences with multiple RC embeddings because multiply embedded RCs are very rare in the human corpus. The sentences encompassed the following structures: transitive/intransitive simple sentences with/without subject, intransitive/transitive subject relative clauses, and transitive object relative clauses. The three types of relative clauses (SRCs with transitive and intransitive verbs and ORCs) could modify either main clause subjects or objects. The script selected words within a given category at random, with equal probability for each word. As with other SRNs, there were very few word units in the model, and all words in a category (such as animate nouns, transitive verbs) were equally frequent as the other words in the category.

**Table 4:** **Finite state grammar with corpus-based bigram transitional probabilities**.

**S → subNP + VP (0.7) / VP (0.3) (meaning that 70% of sentences had a subject NP and 30% were pro-drop sentences, without a subject NP)**	
**VP → VI (0.21) / VT + objNP (0.79) (i.e., 21% intransitive verbs and 79% transitive verbs with direct object NPs)**	
**subNP → aniN(0.5) / inaN (0.34) / subRC (0.16)**	**objNP → aniN(0.18) / inaN (0.73) / objRC (0.09)**
subRC (modifying matrix subject)	objRC (modifying matrix object)
→ SRC_VI (0.11):	→ SRC_VI (0.26):
VI + DE + aniN(0.2)/ inaN(0.8)	VI + DE + aniN(0.31)/ inaN(0.69)
→ SRC (0.58):	→ SRC (0.47):
(0.35) VT + aniN + DE + aniN(0.47)/ inaN(0.53)	(0.29) VT + aniN + DE + aniN(0.53)/ inaN(0.47)
(0.65) VT + inaN + DE + aniN(0.87)/ inaN(0.13)	(0.71) VT + inaN + DE + aniN(0.45)/ inaN(0.55)
→ ORC (0.31):	→ ORC (0.27):
(0.88) aniN + VT + DE + aniN(0.06)/ inaN(0.94)	(0.80) aniN + VT + DE + aniN(0.11)/ inaN(0.89)
(0.12) inaN + VT + DE + aniN(0)/ inaN(1)	(0.20) inaN + VT + DE + aniN(0)/ inaN(1)

S, sentence; NP, noun phrase; VP, verb phrase; VI, intransitive verb; VT, transitive verb; subNP, subject noun phrase; objNP, object noun phrase; aniN, animate noun; inaN, inanimate noun; subRC, subject-modifying relative clause; objRC, object-modifying relative clause; SRC_VI, subject relative clause with intransitive verb; SRC, subject relative clause with transitive verb; ORC, object relative clause; DE, relative clause marker.

#### Model assessment

The model performance was evaluated via Grammatical Prediction Error (GPE), which has been shown to relate well to behavioral measures such as reading times and grammaticality judgments (Christiansen and Chater, 1999). The measure is based on the idea that, because the current output activation is generated by the model reflecting the context in the previous time ticks, the model should activate what is expected/grammatical and should not activate what is ungrammatical, as defined by the training corpus. The GPE therefore incorporates the concepts of “hits” (sum of activation in grammatical units) and “false alarms” (sum of activation in ungrammatical units), with “misses” (sum of insufficient activation of grammatical units), as described in Christiansen and Chater (1999) and shown below.

$$\text{GPE}=1-\frac{\text{hits}}{\text{hits}+\text{false alarms}+\text{misses}}$$

The misses are derived from the difference between the actual output activation from the target activation, based on the transitional probabilities in the training corpus. For example, the target activation of the units representing VT (a transitive verb) at the sentence initial position should sum up to about 0.3 because that value is the sum of all the sentence types with a sentence-initial VT: first, there is 30% chance that the sentence starts as a pro-drop sentence, which is in turn 79% likely to be composed of a transitive verb with an object noun phrase, yielding 0.3 × 0.79 = 0.24. Second, a sentence-initial VT may start an SRC, with a probability of 0.7 × 0.16 × 0.58 = 0.065. Combining the probabilities of the two situations (0.24 + 0.065), we should obtain a target activation for the sentence-initial VT of around 0.3. If in this example the sum of the total output activation in the VT units is actually 0.26, then the misses should be 0.04. Inside the sentence, the target activation distribution was affected by prior context. For example, following the sentence-initial VT, grammatical continuations were aniN (animate noun), inaN (inanimate noun), VT, and VI (intransitive verb). If the next word was an aniN, the VT+aniN fragment could be the start of a) a pro-drop simple sentence with an animate patient (0.3 × 0.79 × 0.18 = 0.043) or b) a subject-modifying SRC (0.7 × 0.16 × 0.58 × 0.35 = 0.023). The total probability of VT + aniN occurring in the whole training corpus was 0.043 + 0.023 = 0.066 but the probability of aniN following VT should be weighted among all four possible continuations. That is, summing up the probabilities of sentence-initial VT + aniN (0.066), VT + inaN (0.3 × 0.79 × 0.73 + 0.7 × 0.16 × 0.58 × 0.65 = 0.215), VT + VT (0.3 × 0.79 × 0.09 × 0.58 = 0.012), VT + VI (0.3 × 0.79 × 0.09 × 0.11 = 0.002) in the corpus, the total probability of sentences starting with an VT should be around 0.30, as calculated above. Among all four legal continuations, the relative proportion of aniN appearing after VT was 0.066/0.30 = 0.22, which should be the summed target activation for all the units representing aniN. The same procedure applied to the other continutations.

It should be noted that overestimation was also implicitly penalized due to the fact that dislocated activation found in some units means insufficient activation in some other units because the total output activation sums to around 1.

The GPE ranges from 0 to 1, with 0 being perfectly accurate in predicting the grammatical categories of the current word based on prior context (that is, 0 error) and 1 being completely incorrect in doing so.

GPEs reflect the prediction error of the next word based on the cumulative context (e.g., the GPE of Word 3 is directly affected by Word 2, which in turn is affected by Word 1), and therefore may implicitly simulate the spillover effect observed in human reading patterns, where reading of one word may be affected by properties of prior input.

#### Testing

Ten networks with different random initial weights, ranging from 1 to -1 with the mean of 0, were created and trained on the 10,000 sentences in the training corpus. These 10 networks simulated the role of “participants” in empirical studies, each of whom may have come from different backgrounds with varying prior experiences and skills. The models were trained on one pass through the training set and were tested using novel test sentences (sentences not contained in the training set) that allowed us to assess the major types of relative clauses that have been investigated in the comprehension literature. There were 16 different types of test sentences, each with 10 tokens, for a total of 160 test sentences. The 16 sentence types were obtained from crossing factors to yield the relative clause types shown in Table 3: two modification positions (main clause subject, object) x relative clause type (SRC vs. ORC) × head noun animacy (animate, inanimate) and RC noun animacy (animate, inanimate). Intransitive SRCs (also shown in Table 3) were not included in the test set because there are no human reading time data in the literature. The GPE scores were calculated at each word in the critical RC region, the head noun, and the word after the head noun.

### Results

In the sections below, we first present results of the model performance at each modifying position (main clause subject or object), noting the general relationships to human data. Then in later sections we present a detailed comparison between the model GPEs and the results of specific experiments. Statistical analyses were conducted with mixed effect models with maximal random effects of participants and items using the lme4 packages in R. Significance values were estimated by likelihood ratio test as suggested in Barr et al. (2013).

#### Subject-modifying RCs

**Figure 3:**
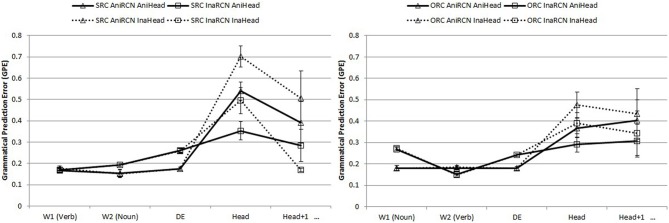
**Word-by-word GPEs of subject-modifying SRCs (left panel) and ORCs (right panel) in the critical RC region, the head, and next word after the head**.

Figure 3 presents the mean GPEs of SRCs and ORCs at each of five word positions comprising a sentence-initial relative clause, a head noun, and the next word. Statistical analyses were conducted to examine three effects—RC type, RC noun animacy, and head noun animacy—along with their interactions. Note that there are no effects of head noun animacy through the first three words, because this factor does not appear in the sentence until the head noun is reached at the fourth word position. The head animacy effects also illustrate a crucial effect of GPE calculations. Before the head noun was encountered, only the effects of RC type, embedded noun animacy, and their interaction were factored into the analyses from Word 1 to DE. The effect of head animacy and its interactions with other two factors were considered only at the head and head + 1 positions.

The most important result for these sentences is the contrast in difficulty at the head position for SRCs (left graph) and ORCs (on the right). As we noted in Tables 2, 3 SRCs modifying a main clause subject are more ambiguous than ORCs: a sentence-initial verb might be the start of an SRC, but competitor pro-drop structures (simple sentences without an overt grammatical subject) are much more common. Sentence-initial object relatives are comparatively less ambiguous, and the effects these different amounts of ambiguity are clear in the model, with reliably higher GPEs for subject relatives than object relatives at the head position (detailed analyses at each word position are given below). Thus, even though sentence-initial SRCs with transitive verbs are more frequent than ORCs at a ratio of almost 2:1, SRCs are substantially harder for the model. This result replicates major findings in the comprehension literature (Hsiao and Gibson, 2003; Gibson and Wu, 2013) and shows how the model's behavior on a particular structure is not simply a reflection of the frequency of that structure. We will elaborate these points after describing the effects at each word position.

***Word-by-word analyses.*** At W1 (RC object for SRCs, RC verb for ORCs), both main effects of RC type (β = −0.009, *SE* = 0.003, *t* = −3.08, *p* = 0.002) and animacy of the RC noun (β = 0.090, *SE* = 0.008, *t* = 11.69, *p* < 0.001) were significant. SRCs were easier than ORCs, and RCs with animate embedded nouns were easier than those with inanimate embedded nouns. An RC type x animacy of RC noun interaction was present (β = −0.093, *SE* = 0.008, *t* = −11.24, *p* < 0.001), primarily because ORCs with inanimate RC nouns were the hardest among all conditions.

At W2, the RC type main effect showed an SRC advantage (β = −0.031, *SE* = 0.013, *t* = −2.34, *p* < 0.001). The main effect of embedded noun animacy was such that RCs with inanimate embedded nouns were easier than those with animate embedded nouns (β = −0.033, *SE* = 0.003, *t* = −10.70, *p* < 0.001). An RC type x RC noun animacy effect existed (β = 0.074, *SE* = 0.012, *t* = 6.39, *p* < 0.001), where SRCs were particularly hard with inanimate RC nouns.

At the RC marker DE, no RC type main effect existed. The RC noun animacy effect remained (β = 0.063, *SE* = 0.007, *t* = 8.71, *p* < 0.001), where RCs with animate embedded nouns were easier. A significant interaction effect of RC type x embedded noun animacy was present (β = 0.022, *SE* = 0.007, *t* = 3.05, *p* = 0.008). SRCs with inanimate RC nouns were particularly hard.

At the head, a main clause transitive or intransitive verb, head animacy was added as a factor in the analysis. All three main effects were significant. The RC type effect showed ORC advantage (β = 0.017, *SE* = 0.052, *t* = 3.32, *p* = 0.001), different from earlier in the RC region. Animacy of the embedded noun stayed significant (β = −0.077, *SE* = 0.028, *t* = −2.78, *p* = 0.043), with inanimate embedded nouns being easier. Head animacy was also reliable (β = 0.011, *SE* = 0.031, *t* = 3.57, *p* < 0.001), where inanimate headed-RCs yielded higher error. Interaction effects were not significant.

At the head + 1 position, no main effects or interactions were present.

### Discussion

Beyond the greater difficulty of SRCs compared to ORCs at the head noun, we observed several other reliable effects in RCs modifying main verb subjects. First, we observed a tendency of early difficulty for RCs with inanimate embedded nouns. Encountering an inanimate noun early in the sentence, a position where animate nouns usually appear as an agent and the grammatical subject, yielded high error. Second, there was an effect of head noun animacy at the head noun, such that animate-headed RCs yielded lower error, reflecting the high frequency of animate nouns being at the main clause subject position in the training corpus. This result has been attested in other corpus data (Wu, 2009) and behavioral studies (Wu et al., 2012).

Third, differences between the two RC types changed over the course of the sentence. ORCs initially yielded higher error rates than SRCs, mostly driven by high error for the (unusual) sentence-initial inanimate noun in ORCs. That is, the performance of the model here shows that simple sentences were initially a competitor (error is high for inanimate nouns sentence-initially, because in the more common simple sentences, sentence-initial nouns are animate). These results are compatible with the results of reading time studies that manipulated noun animacy (Wu et al., 2012). However, other empirical data that found an ORC advantage also exist [e.g., Hsiao and Gibson, 2003; Gibson and Wu, 2013; cf. (Lin and Bever, 2006), for non-significant differences]. As one reviewer noted, GPEs at the sentence-initial verb in SRCs were no larger than GPEs at the sentence-initial noun in ORCs, despite the fact that sentence-initial nouns are much more common than sentence-initial verbs (a 70:30 ratio). The reason may be attributed to the prevalence of verb-starting sentences (i.e., pro-drop sentences and SRCs) in the training corpus and the rather low number of simple sentences compared to the realistic statistics in the language. Sentences starting with a transitive verb (0.3 × 0.79 + 0.7 × 0.16 × 0.58 = 30%) in the training set occurred almost as often as sentences starting with an animate noun (0.7 × 0.5 = 35%). Sentences starting with an inanimate noun, however, were much fewer (0.7 × 0.34 = 23.8%) and thus generated higher error. The error triggered by noun-starting sentences as reflected in transitional probabilities averaged across animate and inanimate nouns is 29.5%, even lower than the 30% for transitive verb-starting sentences. The lower errors in SRCs than ORCs may have been the reflection of such subtle difference in the transitional probability. However, at the same time, this may reflect lower sensitivity of the model to sentence-initial variation, in the absence of prior context, or to the comparatively low number of simple sentences to RCs in the training set, which may have affected sentence initial predictions more than in later regions where prior context is available. Future work with a more varied corpus and more training may clarify this result.

At the RC marker DE, the SRC advantage disappeared and became the opposite pattern at the head: ORCs yielded lower error than SRCs. This effect is consistent with many prior studies in Mandarin that found lower reading times for ORCs (e.g., Hsiao and Gibson, 2003; Lin and Garnsey, 2011; Gibson and Wu, 2013). Importantly, this higher error for SRCs over ORCs appears even though SRCs are more frequent: the model's performance does not simply reflect RC frequencies but also the frequency of competitors and neighbor structures. Next, we look at the model performance on object-modifying RCs, which are more constrained by previous context.

#### Object-modifying RCs

**Figure 4:**
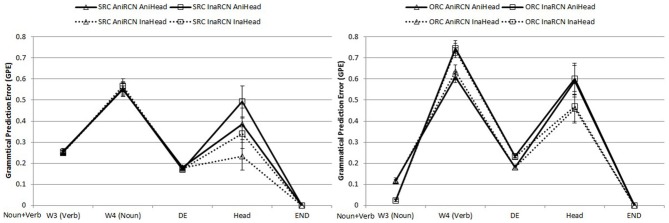
**Word-by-word GPEs of object-modifying SRCs (left panel) and ORCs (right panel) in the critical RC region, the head, and next word after the head**. All sentences began with a Noun + Verb sequence, not shown in the graphs.

To facilitate comparisons across conditions, all test sentences for object-modifying RCs began with an animate main clause subject noun and a transitive verb. As the sentence unfolded to the main clause object position, possible grammatical continuations were more limited than in the sentence-initial RCs reviewed above, owing to the greater preceding context in object-modifying items. Figure 4 shows the trajectory of GPE values for SRCs and ORCs in the RC region, head, and the end-of-sentence marker.

Examination of Figure 4 reveals a general pattern of ORCs being harder than SRCs, in contrast to the subject-modifying relative clauses in Figure 3, for which ORCs were initially slightly harder, and then SRCs were substantially harder after the head noun. This result is also found in the human comprehension literature: Lin and Bever (2006) found longer reading times for object-modifying ORCs than SRCs. Due to the initial unusual word order of two consecutive verbs (main clause verb and RC embedded verb), SRCs are disambiguated early, yielding a small amount of early difficulty but later being easier, because this conjunction of two verbs removes competitors other than an SRC. By contrast, ORCs remain ambiguous: the first word in ORCs in combination with the matrix clause context (the N V N order) created a strong bias toward a simple sentence, which was hard to revise even at the end.

***Word-by-word findings***. Here we call the starting word of an object-modifying RC “W3” (RC verb for SRCs, RC subject for ORCs) because it was preceded by the main clause subject and verb and therefore was the third word in the sentence. At this word, the main effect of RC type indicated that SRCs were significantly harder than ORCs (β = 0.137, *SE* = 0.017, *t* = 7.96, *p* < 0.001). Animacy of the RC embedded nouns had a significant effect too (β = −0.094, *SE* = 0.006, *t* = −16.30, *p* < 0.001), where the inanimate RC nouns induced lower GPEs. The interaction between RC type and embedded noun animacy was significant (β = 0.090, *SE* = 0.008, *t* = 11.16, *p* < 0.0001). ORCs appeared to have a larger GPE difference between the ones with animate RC nouns (higher) and the ones with inanimate RC nouns (lower), whereas the two kinds of SRCs almost had overlapping GPEs.

At W4 (RC object for SRCs, RC verb for ORCs), the previous significant effects reversed: SRCs were now easier than ORCs (β = −0.072, *SE* = 0.024, *t* = −2.98, *p* = 0.008) and animate RC nouns instead produced more accurate predictions than inanimate ones (β = 0.118, *SE* = 0.024, *t* = 4.97, *p* < 0.001). Interaction between the two factors was significant (β = −0.109, *SE* = 0.026, *t* = −4.16, *p* < 0.001). ORCs with inanimate embedded nouns had particularly high GPEs.

At the RC marker DE, RC type was non-significant. The RC noun animacy effect was still present (β = 0.051, *SE* = 0.006, *t* = 8.32, *p* < 0.001). Animate RC nouns again were preferred to inanimate ones. Interaction between the two was significant (β = −0.058, *SE* = 0.011, *t* = −5.53, *p* < 0.001).

At the head, the main effect of RC type was significant, with GPEs of ORCs higher than those of SRCs (β = −0.20, *SE* = 0.098, *t* = −2.08, *p* < 0.001). There was also a significant main effect of head noun animacy (β = −0.13, *SE* = 0.032, *t* = −4.12, *p* < 0.001). Inanimate heads were preferred to animate heads. Other effects and interaction were not significant.

At the end-of-sentence maker, the errors went down to nearly zero for all conditions. No effects were present.

### Discussion

Compared to the subject-modifying position, the model performance exhibited an opposite pattern at the object-modifying site: SRCs were easier than ORCs at the head noun. Error rates reflected early effects of low frequency sequences, followed by lower error rates because these low frequency sequences are themselves highly predictive of subsequent input (e.g., the very rare V V sequence in SRCs yields high error, but a V V sequence strongly predicts subsequent input, leading to low error at the next word). Overall, the SRN's performance on object-modifying RCs once again confirmed the model's sensitivity to both the structural and lexical statistics of the target RC structures and those of competing and neighbor structures under the constraining prior context of a main clause subject and verb. The processing advantage of SRCs over ORCs at the head noun resembles the findings in Lin and Bever (2006), which could be a result of the overall higher structural frequency and the lack of competing structures of SRCs at this position. In the next section, we investigate the effect of modifying position by analyzing SRCs and ORCs at both modifying positions together.

#### Contrasting subject vs. object modification

From the separate analyses above of the two modification sites, we observed almost opposite patterns of GPEs at the head. Prior RC studies have assessed RC difficulty at various regions: words inside the RC, at the head, at the head + 1 position, even further down until the end of sentence, or average across the whole sentence. It is very true that the current model showed a dynamic nature in its processing patterns across the RC, the head, and post-head. Due to the head-final order of RCs in Mandarin, the head is considered the first position where the processing pattern between SRCs and ORCs is differentiated. The working memory-based account argues that integration of related elements in an RC (filler and gap) occurs at this point. Prior empirical studies of Mandarin RCs typically found robust effect of RC type starting from the head Therefore, to compare the difficulty between two RC types at two modification positions, we examined the GPEs at the head position. It was not only the position that reflected the accumulated processing difficulty across the RC but also the first position that reflected the head noun animacy effect.

**Figure 5:**
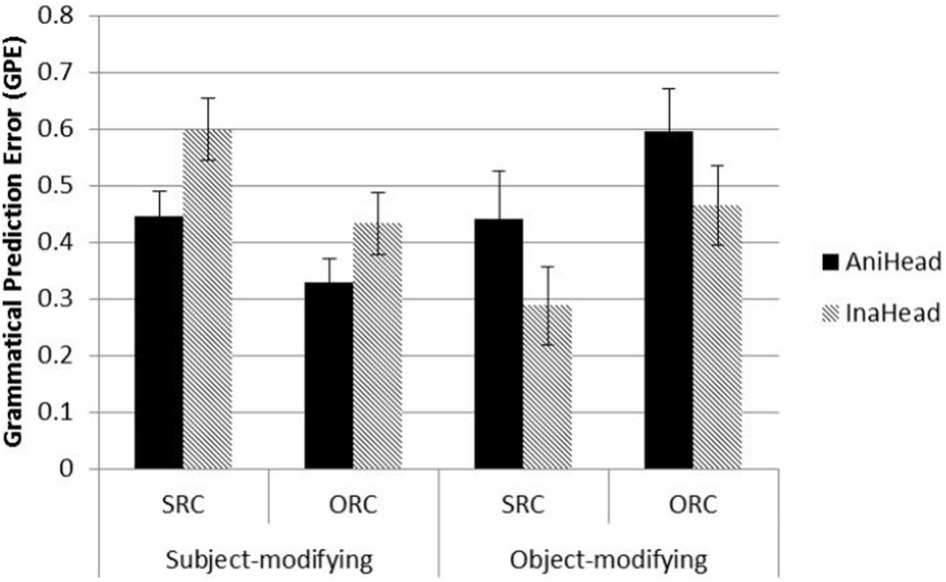
**GPEs of SRCs and ORCs at the head noun**. AniHead = animate head noun, InaHead = inanimate head noun.

Figure 5 displays the average GPEs of SRCs and ORCs at the word following the head noun, across both levels of head noun animacy and modification site. ORCs were easier than SRCs when main clause subjects were modified, whereas SRCs were easier than ORCs modifying main clause objects. Animate heads were preferred to inanimate heads as matrix subjects while inanimate heads were preferred to animate heads as matrix objects.

At this word position, even though there were no significant main effects of RC type, head noun animacy, and modifying position, there were significant interactions of modifying position with RC type (β = 0.37, *SE* = 0.043, *t* = 8.76, *p* < 0.001) and head noun animacy (β = 0.24, *SE* = 0.043, *t* = 5.61, *p* < 0.001). These results suggest that sentential environment (subject vs. object modification) played an important role in the processing the two types of RCs, in combination of the animacy properties of the head. Prior human reading time studies typically have considered only a fraction of the conditions shown, and the conflicting results found in some studies may stem from failures to consider the full pattern of data. Therefore, we examine representative Mandarin RC studies and compare them with our model performance in the next section.

### Comparison with previous mandarin RC reading time studies

In this section we compare the SRN's performance to self-paced reading studies of Mandarin RC processing. As Table 5 shows, there are three major patterns of results in the Mandarin relative clause literature. The first major result is that in studies of subject-modifying relative clauses, ORCs are easier than SRCs, a reverse of the typical pattern in most other languages. Studies yielding this pattern include Gibson and Wu (2013) and Hsiao and Gibson (2003, though only for multiply-embedded sentences in the latter study. An ORC advantage occurred only at the first two words of singly-embedded sentences and Vasishth et al. (2013) reported instead an SRC advantage at the head using Hsiao and Gibson's singly-embedded materials), Su et al. (2007), Chen et al. (2008), and Lin and Garnsey (2011). The SRN captured the major result of subject-modifying ORCs being easier than SRCs. Compared to subject-modifying ORCs, subject-modifying SRCs yielded reliably higher error rates in the model at the head noun (β = 0.17, *SE* = 0.052, *t* = 3.33, *p* = 0.003) and in an average of DE and the head together (as in Gibson and Wu's analysis), the ORC preference was also robust (β = 0.08, *SE* = 0.023, *t* = 3.60, *p* = 0.002).

**Table 5:** **Comparison of experimental materials and major findings of representative human reading time studies**.

**Major data patterns**	**Experiment conditions**	**Studies**	**Specific manipulations**	**Model replication?**
ORCs easier than SRCs at the head noun and/or nearby words	Only subject-modifying RCs, all nouns animate	Gibson and Wu (2013)	Supportive context	Yes
		Hsiao and Gibson (2003)	Doubly-embedded RCs (ORC advantage only at pre-DE region in singly-embedded RCs)	
		Su et al. (2007)	Aphasic patients	
		Chen et al. (2008)	Memory spans	
		Lin and Garnsey (2011)	Topicalization	
Non-replication of Hsiao and Gibson (SRCs easier than ORCs) replication of Gibson and Wu		Vasishth et al. (2013)	Hsiao and Gibson (2003) materials	No
			Gibson and Wu (2013) materials	Yes
Object-modifying SRCs easier than ORCs	Both subject- and object-modifying (all nouns animate)	Lin and Bever (2006)		Yes
No difference between SRCs and ORCs with preferred animacy configuration	Animacy of RC noun and head noun (all subject-modifying)	Wu et al. (2012)	Contrastive animacy of RC noun and head noun	Yes; model shows small differences where Wu et al. find little or no difference

Many of the articles listed in the top row of Table 5 described the ORC advantage as a general tendency of Mandarin sentence processing and working memory limitations, even though their studies investigated only a subset of RCs, namely RCs modifying main clause subjects and containing entirely animate nouns. This brings us to the second and third patterns in Table 5, which reflect the non-universality of the results in the top row. The second major pattern, reported by Lin and Bever (2006), is the effect of modification position. They found that object-modifying RCs were harder than subject-modifying RCs, reflected in reliable reading time differences at many word positions. On first glance, that result does not seem to correspond to the model's performance, but if we examine the model's behavior in the exact sentence types they tested (all animate nouns), the model and human data look much more similar. For this subset of conditions, Lin and Bever's RC-type effect was replicated in our model, with lower GPEs for SRCs compared to ORCs at several word positions (pre-DE: β = −0.04, *SE* = 0.016, *t* = −2.54, *p* = 0.009; head: β = −0.20, *SE* = 0.025, *t* = −8.08, *p* < 0.001). We also roughly replicated their main effect of modifying position (subject-modifying easier) at the pre-DE region (W1 + W2) (β = −0.18, *SE* = 0.016, *t* = −11.26, *p* < 0.001), and also at the head (β = −0.22, *SE* = 0.025, *t* = −8.81, *p* < 0.001). Interactions were significant at these two positions too (pre-DE: β = −0.06, *SE* = 0.021, *t* = −2.84, *p* = 0.005; head: β = 0.38, *SE* = 0.036, *t* = 10.51, *p* < 0.001). When considering each modifying position separately, Lin and Bever did not find any RC type difference for subject-modifying RCs but a significant difference at DE and head for object-modifying RCs. Our model replicated the strong effect of SRC advantage at the object-modifying position (β = −0.20, *SE* = 0.025, *t* = −8.16, *p* < 0.001) that Lin and Bever (2006) found.

The third major pattern is the effect of noun animacy on RC processing, exemplified by Wu et al's. (2012) manipulation of both head and RC noun animacy within subject-modifying relative clauses. Their study did not fully cross head and RC noun animacy and included only contrastive animacy conditions (one noun animate and one inanimate). They found that in the preferred animacy configuration (in which the animate noun was the agent of the RC verb and the inanimate noun the theme), SRCs and ORCs didn't differ in processing difficulty. However, with the dispreferred animacy configuration (inanimate agents, animate patients), ORCs (such as the Mandarin equivalent of *The hiker that the rocks crushed*) were read particularly slowly and an SRC advantage emerged. Our model performance did show such SRC preference for these unusual animacy RCs early at W1 (β = −0.09, *SE* = 0.008, *t* = −11.12, *p* < 0.001) and later at DE (β = −0.07, SE = 0.007, *t* = −10.04, *p* < 0.001), but the ORC became easier at the head (β = 0.41, *SE* = 0.053, *t* = 7.68, *p* < 0.001). For RCs with the preferred animacy configuration, SRCs showed early advantage at W1 (β = −0.01, *SE* = 0.006, *t* = −2.18, *p* = 0.03) but later switched to ORC advantage at DE (β = 0.08, *SE* = 0.011, *t* = 07.25, *p* < 0.001) and switched back at the head (β = −0.12, *SE* = 0.050, *t* = −2.48, *p* = 0.02) but the effect was rather reduced compared to the disfavored animacy configurations. Furthermore, when considering all possible animacy configurations (i.e., AniRCN + AniHead, InaRCN + AniHead, AniRCN + InaHead, InaRCN + InaHead) rather than only the two that Wu et al. (2012) investigated, we found no difference between the favored configurations, namely animate-headed SRCs and inanimate-headed ORCs, at every word except for W1 (β = −0.06, *SE* = 0.011, *t* = −5.28, *p* < 0.001). Thus, the model shows comparatively small differences in the same conditions that Wu et al. find little or no difference in reading times.

In sum, the model captured major patterns of comprehension difficulty across several empirical studies, despite the fact that these studies are often thought to conflict with one another. The model's performance suggests that the inconsistencies in the literature are more apparent than real and stem from different experimental materials used in the experiments, which focus on a small subset of relative clause and animacy types. Whereas it is impossible to manipulate all relevant factors within a single self-paced reading experiment, the current SRN model could incorporate 16 types of test sentences in a 2 × 2 × 2 × 2 design. The model data present a more comprehensive picture, in which RC type, modifying position, head noun animacy, and RC noun animacy all have an effect. These data suggest that relative clause difficulty in Mandarin depends on a complex interplay of probabilistic constraints from animacy and other information gleaned from prior experience. Thus, contra many claims in the literature (e.g., Hsiao and Gibson, 2003; Lin and Bever, 2006; Gibson and Wu, 2013; Vasishth et al., 2013) none of the empirical results warrant broad conclusions about ORCs or SRCs being universally easier or harder.

## General discussion

In this paper, we presented an SRN simulation of the processing of Mandarin relative clauses. We had two related goals. First, we wanted to use the model to investigate issues that are difficult to test in human experiments. We suspected that controversies in the empirical literature stemmed at least in part from complex interactions among a number of factors such that when researchers designed materials tapping different subsets of the factors, different results obtained. Constraints on human studies, such as biases or priming effects that arise when comprehenders encounter many sentences of the same type, typically prevent anything more complex than a 2 × 2 design in sentence processing studies. By contrast, our 2 × 2 × 2 × 2 design presented in 16 test sentence types showed that the four different factors we examined (RC type, modifying position, head animacy, and RC noun animacy) interacted in complex ways in the model. The results from the model closely track reading time patterns from a number of human comprehension studies and suggest that there is no overall SRC or ORC preference in Mandarin. Instead, the results strongly depend on which types of relative clauses are contrasted. In relative clauses with the animacy configurations most commonly investigated in human studies to date, ORCs are easier than SRCs when they modify main clause subjects but the reverse is true when they modify main clause objects, but other patterns are obtained with different animacy configurations, which can be seen most clearly in Figure 5. These results suggest that claims for broad categories of relative clause difficulty in Mandarin are premature at best.

Second, we wanted to use an SRN's ability to generalize over similar items (MacDonald and Christiansen, 2002; Fitz et al., 2011) to investigate how competitor and neighbor interpretations affect RC interpretations. Here our corpus analysis showed that RCs in Mandarin can be highly ambiguous for human comprehenders. When the model was trained on the resulting training set, prediction error varied with all four factors investigated here.

An important component of this second goal was our incorporation of animacy information in the corpus analyses and in the model. Human comprehension patterns clearly show the importance of animacy information in relative clause processing (Wu et al., 2012), and the model also captured these animacy effects, despite having no conceptual information that would typically be used to code distinctions between animate and inanimate entities. These results show the power of the sequential information associated with animacy, that sentences containing animate entities have different distributional patterns in the language than those with inanimate nouns. Chang (2009) made a related argument in his examination of cross-linguistic variation in language production, that learning over the distributional regularities of various animacy configurations in a language is critical for explaining why animacy has different effects in a relatively strict word order language such as English compared with a relatively free word order language such as Japanese—people are learning the sequential information associated with sentences of different types, and the animacy-structure patterns vary as a function of the rigidity of the word order in the language. His results, as well of those of our model, suggest that sequential learning is an important adjunct to conceptual information in accounts of animacy effects in sentence-level language use.

Another important component of our investigation of neighborhood effects is the link between generalization over similar sentences and a topic that initially seems unrelated: the role of working memory in accounts of language comprehension. Because of its generalization over many sentence types, the model shows patterns of difficulty analogous to those in human studies. Critically, it does so in a system in which experience influences the model's computational capacity (and thus its ability to make accurate predictions for upcoming input, MacDonald and Christiansen, 2002). This result contrasts with claims questioning the adequacy of experience-based accounts in RC processing. Levy et al. (in press) and Levy and Gibson (2013) have assessed experience via calculations of a word's surprisal—the conditional probability of that word given prior context. They suggest that surprisal does not correctly predict the full pattern of human relative clause reading times, and they argue that human comprehension difficulty requires supplementing surprisal with an account of human memory burdens, as in Gibson's (1998) Locality theory, in which RC difficulty varies with load in an experience-independent working memory. Given our own results and MacDonald and Christiansen's success in using an SRN to model humans' reading times of English RCs, we think it is premature to reject all experience-based accounts on the basis of failures of particular implementations (particular surprisal instantiations). First, as Frank (2009) notes, the success of Levy and colleagues' surprisal calculation varies with the richness of the prior input. Thus, it may be that a larger or more realistic corpus over which to calculate conditional probabilities would yield a better account of humans' experience and consequently better prediction of reading times. However, we suspect there is a second reason why SRNs can yield different predictions than Levy and colleagues' surprisal results, concerning how context is represented and transformed into predictions. As it has been implemented to date, surprisal is based on an aggregation of past instances, which is used to calculate the conditional probability of upcoming input. By contrast, the SRN is a learning model that compresses and transforms its experience into an internal representation as it learns (Elman, 1990; Tabor et al., 1997; Frank, 2009). As a result, the SRN generalizes over neighboring structures and shows behavior that is not always a sum of instances in the training set, such as when the more frequent subject-modifying SRC sentences yield higher error rates than the rarer subject-modifying ORC sentences. A more direct comparison of SRNs and various instantiations of surprisal should be an important step in better understanding the role of experience in RC processing (see also Frank, 2009). In the meantime, we see no need to complicate our experience-based account with an additional component as in Levy and Gibson's proposal, and indeed we see the success of our SRN as further evidence for the non-independence of experience and computational capacity/working memory (McClelland and Elman, 1986; MacDonald and Christiansen, 2002).

### Future directions

The SRN we used was trained on a set of sentences based on realistic human linguistic experiences, gleaned from a detailed corpus analysis. Although other corpus analyses exist for Mandarin RCs (Hsiao and Gibson, 2003; Pu, 2007; Wu, 2009; Vasishth et al., 2013), our study was unique in its large scale (incorporating many non-RC structures), its hand-coding of animacy across both RC and non-RC sentences, and its use in training the SRN. The combination with the SRN is crucial here because RC processing difficulty in both human and model is not simply a function of the frequency of RCs. We consider the following points important steps to further improve the current model.

The claim that Mandarin RC processing is tied to uncertainty of predictions echoes similar claims for other languages (e.g., Gennari and MacDonald, 2008). The SRN offers important opportunities to more stringently test these claims in future research. For example, to test whether simple pro-drop sentences are truly a competitor for certain RCs, the current model (containing pro-drop sentences) can be compared to one trained on a variant of Mandarin without pro-drop (i.e., replacing all pro-drop sentences in the training set with overt subject variants). We are pursuing these and other model comparisons aimed at elucidating the role of neighbors/competitors in the input. In addition, we intend to manipulate the amount of training, as in MacDonald and Christiansen (2002), to further observe the developmental trends in learning and generalizing. Thus, SRNs can help trace the sources of processing difficulty using methods that are impossible to use with human comprehenders.

Another future endeavor should involve a more accurate portrayal of the rate of RCs and other related structures in Mandarin. For example, the multiple functions of *DE* (see Appendix A), which has been considered a disambiguating cue in RCs can in actuality create ambiguities depending on the semantic context. Structures involving these other *DE* uses should be considered potential competitors. Another typological feature in Mandarin that might be relevant is the possibility of null object (e.g., He saw (the movie).) with supportive context, contrastive to null subject considered in the current model. The effects of null object and its combination with null subject are yet to be explored. Of course models necessarily remain simplifications of the entire linguistic experience (and simplifications of many other dimensions of human cognition). Model expansions therefore should not just cover more data but provide new insight into how people weigh multiple probabilistic constraints during sentence interpretation.

An important feature of our training set was its coding of distributional aspects of lexical meaning, as originally demonstrated by Elman (1990). That is, the model had no explicit semantic representations but it came to distinguish animate and inanimate nouns and transitive and intransitive verbs by virtue of their different distributions in the training set. These fine-grained discriminations were crucial for the model's account of animacy effects in RC processing. These and other studies of distributional semantic effects in SRNs (Elman, 1990; St John and McClelland, 1990) show that there is potential for future work to incorporate other distributional “semantic” effects. There is also potential to make the distributions more natural, thereby capturing some additional ambiguity effects not in the present model. For example, for the current model, all verbs in the training set were either 100% transitive or 100% intransitive, with no optionally transitive verbs such as *eat*, which can occur either with or without a direct object. Optionally transitive verbs add additional indeterminacy, in that a model encountering an optionally transitive verb will be uncertain about an upcoming direct object, an effect which could modulate differences between SRCs (which can have transitive or intransitive verbs) and ORCs (which must have transitive verbs).

## Conclusion

The current study confirmed SRN models as a promising tool in modeling human sentence processing, and, in this particular case, appropriate to examine intricate and complicated dynamics of Mandarin RC processing. The architecture of SRNs allows flexibility in modeling multiple effects in a single model, whereas manipulating a large number of factors human studies is nearly impossible. Mandarin is typologically unique in its conjunction of head-final RCs and head-initial SVO basic word, and yet in some sense the model's behavior looks very similar to that of SRN models of English RCs (MacDonald and Christiansen, 2002; Fitz et al., 2011). That is, the patterns of SRC vs. ORC difficulty are wildly different for the two languages, but in both cases, the models are strongly affected by the balance of RCs, competitors and neighbors. The modeling results suggest that rather than arguments for universal Locality (Gibson, 1998) or universal SRC preference (Lin and Bever, 2006), the real universals in human RC processing are exquisite sensitivity to the statistical regularities of across many different types of input.

### Conflict of interest statement

The authors declare that the research was conducted in the absence of any commercial or financial relationships that could be construed as a potential conflict of interest.

## Appendix A

### Ambiguity and competitor interpretations for mandarin relative clauses

Due to the head-final feature of Mandarin relative clauses and the fact that the relative pronoun DE appears at the end of a relative clause, Mandarin relative clauses contain many temporary ambiguities. Even at the relative clause marker DE, the structure could still be ambiguous because DE has other functions in Mandarin beyond the relative pronoun. Below, we detail some of these ambiguities—strings that could turn out to be a relative clause of some type but could also turn out to be some other kind of structure. In some cases, these structures may conflict with the relative clause interpretation at one sentence position but may at other times facilitate the RC interpretation (be a neighbor).

#### Simple sentences

The dominant Subject-Verb-Object word order in Mandarin creates different amounts of ambiguity for object relative clauses occurring at different main clause positions. For example, an object relative clause modifying a main clause subject begins with the sequence “N-V” (as in 2b) which can be interpreted as a main clause subject and verb (the simple sentence is a temporary competitor), but later regions of the sentence reveal that this initial N-V sequence was instead part of a relative clause. The prevalence of Mandarin Subject-Verb-Object simple sentences could potentially aid the processing of sentences that share word order similarities (MacDonald and Christiansen, 2002; Fitz et al., 2011). Thus, the fact that simple transitive sentences and subject-modifying object relatives initially have the same word order creates first an ambiguity, and then when the simple sentence interpretation is removed by additional input, the simple sentences are a helpful neighbor, in that past experience with simple sentences can help interpretation of these relative clauses.

By contrast, simple transitive sentences are not a competitor or neighbor for subject relative clauses modifying a main clause object (as in 3a). These structures contain a sequence of two verbs early in the sentence, which rules out a simple sentence interpretation of the input. Subject relatives might therefore be somewhat difficult early when the unusual sequence of two verbs is encountered, but overall, these sentences are less ambiguous than the object relatives, for which the simple sentence interpretation can persist over more input.

These examples show that generalization and competition from simple sentences manifest differently in the two main clause positions. These patterns are important because the dominant results in the comprehension literature find that subject relatives in subject-modifying position are harder than object relatives (Hsiao and Gibson, 2003; Gibson and Wu, 2013), while the opposite pattern obtains for object-modifying RCs (Lin, 2006; Lin and Bever, 2006).

#### Pro-drop sentences

One important competitor structure for relative clauses comes from simple main clause sentences in which the subject NP has been omitted. Mandarin is a pro-drop language (meaning that in context, grammatical subjects are omissible; Li and Thompson, 1981), so that simple sentences such as “VT-Object” exist as complete sentences in the language. These pro-drop sentences are temporary competitors for subject-modifying subject relatives, which have the sequence “VT-Object-DE-Subject.” As more input comes in to disambiguate the structure, the comprehender may recover from the initial difficulty and be aided by this resemblance in word order with pro-drop sentences. Such facilitation effect for subject relatives may be even more pronounced and activated sooner at the main clause object site. The unusual word order of two verbs in a row after the main clause subject creates short-term difficulty but meanwhile also provides a reliable cue for a subject relative clause and facilitates processing of the rest part.

#### Subject relative clauses with intransitive verbs

Subject relatives with intransitive verbs are rarely examined in prior empirical studies. Structurally, they resemble subject relatives with transitive verbs, with the lack of a direct object. Thus, experiences with one structure could be likely transferable to the other.

#### Other structures with DE

The surface word order of Mandarin relative clauses is identical to many other structures that also have the particle DE. In addition to serving as a relativizer in a relative clause, DE is also a marker for adjectives and adverbs and also appears in possessives and appositives or simply as a phrase-final particle. With these many functions of DE, which is sometimes considered the disambiguating point for a relative clause, the relative clause structure could still be ambiguous after DE and permits various interpretations; the only definitive disambiguation is discourse context. For example, in the phrase “respect teacher DE parents,” the DE could be interpreted as a relativizer of a subject relative: “the parents that respect the teacher,” or as a possessive marker in a nominalized VP gerund: “respecting the teacher's parents.” The appositive structure with DE also shares identical word order with a subject relative, such as in “respect teacher DE policy,” which could be either interpreted as “the policy that respects teacher” (RC interpretation) or “the policy about respecting teachers” (appositive interpretation).

Because the SRN in Study 2 will not code for semantics, except for animacy, we focus on only the relativizer use of DE. This represents a simplification compared to the full natural language.

Summarizing from above, we can see that these relevant structures, some of which may well surpass the frequency of relative clauses in comprehenders' linguistic experiences, impose influences on the processing of both the typical subject relative clauses with transitive verbs and object relative clauses, at different main clause site.

## Appendix B

**Table B1:** **Tgrep2 search patterns for structures in Study 1**.

Simple sentences, transitive	/^IP/<(/^NP-SBJ/!</^-NONE-/!<<DEC)<(/^VP/<<VV<<(/^NP-OBJ/!</^-NONE-/!<<DEC))!>>/^IP/
Simple sentences, intransitive	/^IP/<(/^NP-SBJ/!</^-NONE-/!<<DEC)<(/^VP/<<VV!<</^NP-OBJ/)!>>/^IP/
Pro-drop sentences, transitive	/^IP/<(/^NP-SBJ/</^-NONE-/)<(/^VP/<<VV<<(/^NP-OBJ/!</^-NONE-/!<<DEC))!>>/^IP/
Pro-drop sentences, intransitive	/^IP/<(/^NP-SBJ/</^-NONE-/)<(/^VP/<<VV!<</^NP-OBJ/)!>>/^IP/
SRC, transitive	/^NP-SBJ/ (or /^NP-OBJ/ for object-modifying)<<(/^IP/<(/^NP-SBJ/<(/^-NONE-/!<^*^PRO^*^!<^*^pro^*^)<(/^VP/<<VV<< (/^NP-OBJ/!</^-NONE-/))$(DEC../^NP/))
SRC, intransitive	/^NP-SBJ/(or /^NP-OBJ/ for object-modifying)<<(/^IP/<(/^NP-SBJ/<(/^-NONE-/!<^*^PRO^*^!<^*^pro^*^)<(/^VP/<<VV!<</^NP-OBJ /))$(DEC../^NP/))
ORC	/^NP-SBJ/(or /^NP-OBJ/ for object-modifying)<<(/^IP/<(/^NP-SBJ/!</^-NONE-/)<(/^VP/<<VV<<(/^NP-OBJ/</^-NONE-/))$(DEC../^NP/))
